# Bone health in rural Australia: a mixed methods study of consumer needs

**DOI:** 10.1007/s11657-023-01333-8

**Published:** 2023-10-14

**Authors:** Alicia R Jones, Belinda Garth, Catherine Haigh, Peter R Ebeling, Helena Teede, Amanda J Vincent

**Affiliations:** 1 Monash Centre for Health Research and Implementation, School of Public Health and Preventive Medicine, Monash University, Locked Bag 29, Clayton, Vic, Melbourne, 3068 Australia; 2 Department of Endocrinology, Monash Health, Melbourne, Australia; 3 Monash Rural Health Gippsland, Monash University, Traralgon West, Australia; 4 Department of Medicine, School of Clinical Sciences, Monash University, Melbourne, Australia; 5 Monash Partner’s Academic Health Science Centre, Victoria, Australia

**Keywords:** Osteoporosis, Consumer preference, Qualitative research, Rural health services, Rural health

## Abstract

***Summary*:**

This mixed methods study explores osteoporosis among adults living in a regional area of Victoria, Australia. Three major themes emerged from interviews, which reflected the findings of surveys, concerns regarding the adequacy of care in rural areas, a desire for tailored, local care, and a desire for hybrid telemedicine or in-person services.

**Purpose:**

Osteoporosis or osteopenia affects over half of adults aged over 50 years. People living outside major cities in Australia have higher hip fracture rates than people living in cities, along with reduced access to bone densitometry and osteoporosis specialists. This study explores osteoporosis risk factors, knowledge, experiences of and preferences for care in people living in a regional area, to inform development of osteoporosis care programs.

**Methods:**

Adults living in a large non-metropolitan region of Australia were invited to participate in a mixed methods study: a survey (phase 1) followed by semi-structured interviews (phase 2) with triangulation of results. Data collected included osteoporosis diagnosis, risk factors, management, knowledge, preferences for care and experience using telemedicine. Surveys were analysed quantitatively, with linear and logistic regression used to assess factors related to osteoporosis knowledge or satisfaction with telemedicine. Interview transcripts were analysed using thematic analysis by two researchers, with in-depth discussion to identify themes.

**Results:**

Sixty-two participants completed the survey, and 15 completed interviews. The mean (SD) age of survey participants was 62.2 (14.1) years, 57% had a screening test for osteoporosis, and 12 (19%) had a diagnosis of osteoporosis. The mean osteoporosis knowledge score was 8.4 / 19 and did not differ with age, education, or history of osteoporosis. The majority wanted access to more information about osteoporosis but preferred method differed, and the majority preferred in-person medical consultations to telemedicine. Interview participants were aged between 57 and 87 years, and included 8 with osteoporosis or osteopenia. Three major themes emerged: concerns regarding the adequacy of care in rural areas, a desire for tailored local car and a desire for hybrid telemedicine or in-person services.

**Conclusion:**

Gaps exist in rural osteoporosis care, including knowledge, screening and management. People have differing experiences of care, access to services and preferences for care. High-quality care, tailored to their needs, was preferred. Improving osteoporosis services for regional Australia will require a flexible, multi-faceted approach, addressing needs of the local community and providers.

**Supplementary Information:**

The online version contains supplementary material available at 10.1007/s11657-023-01333-8.

## Introduction

Approximately two-thirds of adults aged over 50 years have either osteoporosis or osteopenia, contributing to an increased risk of fracture [1, 2]. Osteoporosis and the associated fractures cause significant morbidity and mortality, with estimated morbidity greater than many other non-communicable diseases, such as ischaemic stroke or chronic obstructive airway disease [3].

Geographic discrepancy in osteoporosis care is evident, with variation in availability of DXA, use of treatment and access to specialist services both across and within countries [4–8]. A systematic review suggested lower fracture risk in rural compared to urban areas of higher income countries; however, this pattern was not seen in lower income countries and may be country- or region-specific [9]. Similar disparities exist in rural areas for other chronic conditions globally [10].

In Australia, despite similar rates of self-reported osteoporosis in rural and urban areas, there are higher rates of hip fractures in rural areas [5, 11]. The reasons for geographical discrepancy are unclear, and likely multifactorial, with patient-related factors, clinician-related factors and system factors, such as variation in health-related behaviours, osteoporosis knowledge and availability of bone densitometry or bone health specialists [11, 12].

In 2019, the Australian Government launched a National Strategic Action Plan for Osteoporosis, prioritising actions to improve osteoporosis management [13]. A National Consumer and Community Forum identified key areas for improvement including increasing community awareness, improving risk factor identification, diagnosis and fracture management.

Approximately one third of Australians live outside major cities [11]. However, people in rural areas are less likely to be included in medical research [14, 15]. Stakeholder engagement is a key component of designing and implementing models of care [16]. Communities and clinicians living outside major cities have distinct experiences and preferences for healthcare, which are important to capture in order to improve care delivery and outcomes [12, 17].

Our study aimed to explore the unique needs and perspectives of people living in a large regional area of Australia, in regard to osteoporosis. We sought to explore knowledge gaps, risk factors, preferences for care and barriers to care, in order to inform development of osteoporosis models of care.

## Methods

This mixed methods study design included a quantitative survey (phase 1), followed by qualitative semi-structured interviews (phase 2), with triangulation of results. The survey provided information on osteoporosis prevalence, risk factors, management and knowledge, while interviews provided further insight into perceptions, preferences and experiences of osteoporosis care. Data from the survey and interviews will be used to inform the development of osteoporosis care programs.

### Participants and setting

Adults aged ≥ 18 years living in Gippsland, a large region of Victoria, Australia were invited to participate. This region was chosen because it had the highest rates of hip fractures (a fracture indicative of osteoporosis) in Victoria in 2012–2013 [5]. We included both people with and without osteoporosis, because osteoporosis is often undiagnosed, and the perspectives of those without osteoporosis are an important aspect to prevention. Exclusion criteria were aged < 18 years, or non-English speaking. Participants were recruited via advertisement at local community groups, social media, local radio stations and local newspapers, as well as word of mouth.

### Phase 1: survey

The quantitative survey involved a 38-question anonymous survey, completed online or via mailed hard copy distributed at community meetings (Online resource 1). The survey was developed by the research team, pilot tested with members of the community and refined based on feedback. The survey contained questions regarding demographic details, residential location (based on the Modified Monash Model level of remoteness [18]) osteoporosis diagnosis, management and osteoporosis risk factors, osteoporosis knowledge (using the Osteoporosis Knowledge Assessment Tool, (OKAT)) [19] and preferences for managing osteoporosis. Due to the expansion of telemedicine across Australia during the COVID-19 pandemic, specific questions were included regarding experience of telemedicine. An explanatory statement was provided at the start of the survey, and consent was given electronically (online) or implied by return of the survey (hard copy). We initially aimed to recruit at least 100 participants; however, the recruitment was difficult, and we closed the survey after 18 months (available October 2020–June 2022).

Surveys were analysed quantitatively, with categorical data reported as number (%) and continuous data as mean (standard deviation). One question was omitted from the OKAT as it did not align with current Australian guidelines, and thus the potential total score was 19 instead of 20 [20]. Linear regression was used to assess the relationship of age, educational status, residential location or a diagnosis of osteoporosis, and osteoporosis knowledge. Logistic regression was used to assess the relationship between age, educational status and residential location, and satisfaction with telemedicine. A significance level of *p* < 0.05 was used. REDcap electronic data capture tools hosted at Monash University were used for data collection and management, and Stata V15 (StataCorp LLC, Tx, USA) was used to analyse data [21, 22].

Although the survey was anonymous, participants could record their email address to be contacted to participate in an interview. To encourage participation, by leaving an email address, participants entered a draw to win one of five $75 AUD gift vouchers, which was selected via a random number generator. The email addresses were stored separately, so they could not be linked to the survey data.

### Phase 2: semi-structured interviews

Semi-structured telephone interviews were conducted between November 2020 and July 2021. All participants who completed the survey and expressed interest in an interview were contacted via email to confirm interest and determine availability to participate. Of the 23 who expressed interest, 16 responded; one was not eligible as they lived mainly in a metropolitan area, the remaining 15 agreed to an interview and an explanatory statement was provided to these participants. Verbal consent was obtained by telephone prior to commencing the interview. Participants were reimbursed for their time with a $50 AUD gift voucher.

Interviews were conducted by an Endocrinologist with experience in osteoporosis and research (AJ) and lasted 20–25 min. The interview schedule (Online resource 2) was developed by the research team and informed by previous literature [23–25]. Demographic details were collected prior to commencing the interview, and the interview schedule differed based on whether the participant had a diagnosis of osteoporosis or osteopenia or not. Interview topics included osteoporosis knowledge, osteoporosis diagnosis and current management, their ‘ideal’ care program (participants without osteoporosis were asked to imagine that they did have osteoporosis), educational needs and preferences in relation to bone health and use and satisfaction with telemedicine.

Interviews were audio-recorded and transcribed verbatim by an independent transcribing service. Deidentified transcripts were analysed by an Endocrinologist / researcher with experience managing osteoporosis (AJ) and an experienced qualitative researcher (BG). Thematic analysis was used with an inductive approach informed by our research question and review of the literature, to draw out shared meaning and patterns across the data [26]. Both researchers independently coded three transcripts and met to review codes and reach agreement on interpretation of data and coding. AJ then coded the remaining transcripts, followed by review of these by BG. After assigning initial codes, these were grouped into broader themes, with in-depth discussion between researchers to achieve consensus. Data were informed by a range of participant viewpoints and held sufficient information power to develop new knowledge; data saturation rarely truly occurs in qualitative research of this type [27, 28]. The Dedoose platform was used to store and organise data.

## Results

### Phase 1: surveys

Seventy participants attempted the survey, and after excluding those who did not consent to continue or did not complete any questions, 62 participants were included in the final analysis. As recruitment involved various strategies across local and social media, response rate could not be determined. Demographic data and osteoporosis risk factors are shown in Table 1. Participants were predominantly female, aged from 31 to 87 years, and most had completed higher education; 52 were born in Australia (8 born in Europe / UK, 1 born in USA, 1 born in south-east Asia) and most used technology (smart phone, tablet or computer) multiple times per week. No participants identified as Indigenous or resided in remote locations.

**Table 1:** Demographic details and osteoporosis risk factors of survey participants. *N* = 62 unless otherwise stated

Age, years, mean (SD) (*n* = 56)		62.2 (14.1)
Female, *n* (%)		58 (93.6%)
Australian born, *n* (%)		52 (83.9)
Education, *n* (%)	Did not complete high school	13 (21.0)
	High school completion	5 (8.1)
	Vocational qualification / diploma	19 (30.6)
	Bachelor degree	12 (19.4)
	Higher degree (Master’s or PhD)	13 (21.0)
Residential location (Modified Monash Model) *n* (%) (*n* = 59)	1. Metropolitan	6 (10.2)
	2. Regional centre	3 (5.1)
	3. Large rural town	10 (17.0)
	4. Medium rural town	22 (37.3)
	5. Small rural town	18 (30.5)
Smoking status *n* (%)	Never	39 (62.9)
	Former	20 (32.3)
	Current	3 (4.8)
Alcohol intake *n* (%)	None	19 (30.7)
	< 1 standard drink (SD) per day	34 (54.8)
	1-2 SD per day	7 (11.3)
	> 2 SD per day	2 (3.2)
Exercise per week *n* (%) (*n* = 61)	< 75min moderate or 35min vigorous	17 (27.9)
	75-150min moderate or 35-75min vigorous	24 (39.3)
	150-300min moderate or 75-150min vigorous	10 (16.4)
	>300min moderate or >150min vigorous	10 (16.4)
Family history^a^ *n* (%) (*n* = 61)	Yes	14 (23.0)
Comorbidities^b^ *n* (%) (*n* = 61)	0	29 (46.8)
	1	20 (32.3)
	2 or more	13 (21.0)

^a^Family history included presence of parental hip fracture or parental diagnosis of osteoporosis

^b^Comorbidities include: coeliac disease, chronic kidney disease, chronic liver disease, hyperthyroidism, hyperparathyroidism, vitamin D deficiency, rheumatoid arthritis, obesity body mass index > 30, underweight body mass index < 18.5, epilepsy, diabetes (type 1 or type 2), malabsorption, glucocorticoid use for 3 months or longer, early menopause before age 45 years

#### Osteoporosis screening, diagnosis, treatment and knowledge

Slightly over half (57%) of participants reported having a screening test for osteoporosis including dual X-ray absorptiometry (DXA) or heel ultrasound. The majority (*n* = 44, 71%) had one or more risk factor for osteoporosis (either a comorbidity known to increase osteoporosis risk, excess alcohol intake, smoking or family history, but excluding age and sex). However, of those participants with osteoporosis risk factors, 16 (36%) had not had a screening test for osteoporosis.

A diagnosis of osteoporosis was reported by 12 (19%) participants, although 13 (21%) reported having one or more minimal trauma fractures. Of those with a previous fracture, only 5 reported having a diagnosis of osteoporosis. Regarding those participants with osteoporosis, 7/12 (58%) were prescribed an antiresorptive medication currently or in the past, and 2/12 were managed by a specialist.

The mean (SD) OKAT score was low, 8.4 (3.9) /19, and less than half of respondents answered correctly for 11 questions (see Table 2). Using linear regression, participants with osteoporosis or previous fractures did not have higher osteoporosis knowledge (*p* > 0.05), and neither age nor education was associated with OKAT score.

**Table 2:** Osteoporosis knowledge assessment tool answers

Question	Correct answer	Number (%) of correct answers
Osteoporosis leads to an increased risk of bone fractures	True	58 (93.6)
Osteoporosis usually causes symptoms (e.g. pain) before fractures occur	False	28 (45.2)
Having a higher peak bone mass at the end of childhood gives **no** protection against the development of osteoporosis in later life	True	16 (25.8)
Osteoporosis is more common in men	False	46 (74.2)
Cigarette smoking can contribute to osteoporosis	True	40 (64.5)
White women are at highest risk of fracture as compared to other races	True	15 (24.2)
A fall is just as important as low bone strength in causing fractures	True	31 (50)
By age 80, the majority of women have osteoporosis	True	27 (43.6)
From age 50, most women can expect at least one fracture before they die	True	15 (24.2)
Any type of physical activity is beneficial for osteoporosis	False	13 (21.0)
It is easy to tell whether I am at risk of osteoporosis by my clinical risk factors	True	21 (33.9)
Family history of osteoporosis strongly predisposes a person to osteoporosis	True	35 (56.4)
Sardines and broccoli are good sources of calcium for people who cannot take dairy products	True	47 (75.8)
Calcium supplements alone can prevent bone loss	False	41 (66.1)
Alcohol in moderation has little effect on osteoporosis	True	19 (30.7)
A high salt intake is a risk factor for osteoporosis	True	9 (14.5)
There is a small amount of bone loss in the ten years following the onset of menopause	False	6 (9.7)
Hormone therapy prevents further bone loss at any age after menopause	True	16 (25.8)
There are no effective treatments for osteoporosis available in Australia	False	37 (59.7)

#### Osteoporosis care delivery

Table 3 shows preferences for an osteoporosis care program. Although two thirds wanted access to information to learn more about their condition, the preferred method of receiving information differed.

**Table 3:** Preferences for osteoporosis care

		Yes (*n*/%)
Receiving care close to home		51 (82.3)
Seeing a specialist		40 (64.5)
Seeing an osteopath		17 (27.4)
Seeing a physiotherapist or exercise physiologist		38 (61.3)
Seeing a naturopath or complementary practitioner		8 (12.9)
Ability to use telemedicine for appointments		18 (29.0)
Low cost healthcare providers		41 (66.1)
Low cost medications		38 (61.3)
Flexible appointment times		25 (40.3)
Access to information to learn more about my condition		41 (66.1)
Preferred source of information	Online info	21 (33.9)
	Hard copy written	17 (27.4)
	Video	13 (21.0)
	Group education sessions	11 (17.7)
	Individual education	13 (21.0)

The entire cohort, with and without osteoporosis, are included. Those without osteoporosis were asked to imagine that they did have osteoporosis, for this question

**Fig. 1:**
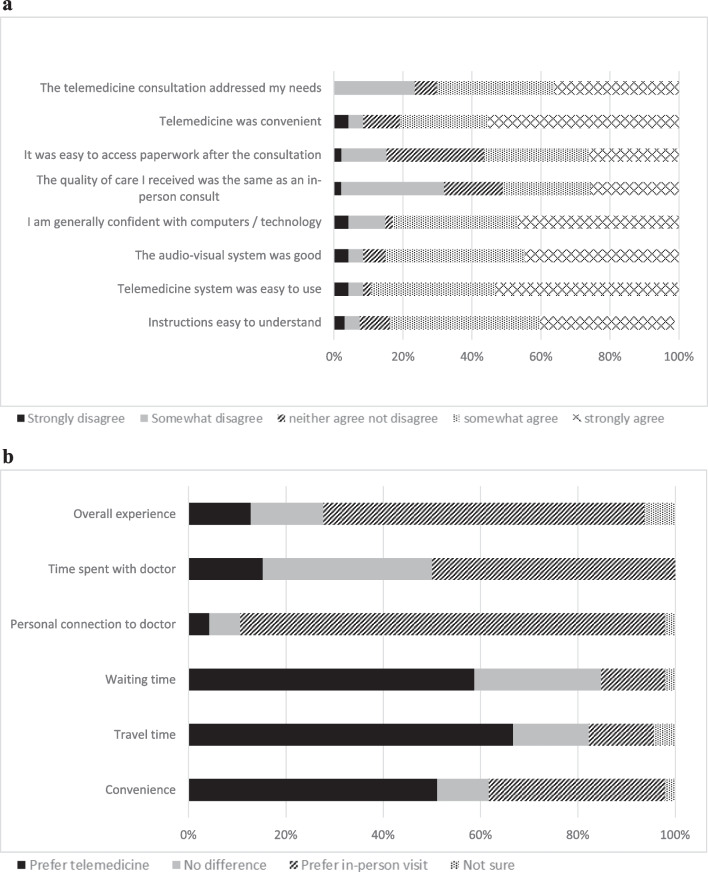
Assessment of telemedicine. **a** Satisfaction with telemedicine. **b** Comparison of telemedicine and in-person appointments

Most participants (*n* = 47, 78.3%) had used telemedicine during the COVID-19 pandemic, whereas prior to the pandemic, only 2 had used telemedicine. The vast majority of these (*n* = 45) were telephone consults. Overall, participants were satisfied with individual aspects of telemedicine (Fig. 1a), and 34 (72.3%) would use telemedicine again. Despite this, the majority (66%) would prefer an in-person appointment if offered the choice (Fig. 1b). Most respondents preferred telehealth regarding waiting time, travel time and convenience. Logistic regression analysis found preference for in-person appointments was not related to age, education level or residential location (all *p* > 0.05). Regarding concerns with telemedicine, 27 (46%) found it difficult to feel connected to their doctor, and 24 (43%) were concerned that they received inadequate treatment via telemedicine. Privacy and technology concerns were less common (11% and 23%, respectively).

### Phase 2: semi-structured interviews

Fifteen telephone interviews were conducted, including eight participants who had osteoporosis or osteopenia (OP) and seven who did not (noOP). The mean (SD) age overall was 70 (8.2) years, while the mean (SD) age of those with osteoporosis was 73.3 (8.8), and of those without osteoporosis was 66.6 (5.7). Fourteen participants were female. Demographic details of interview participants are supplied in the Online Resource 3. Of those with osteoporosis or osteopenia, two were currently treated with denosumab (P1, P2), one with zoledronic acid (P10) and one with testosterone (P12), and three had consulted a specialist (P4, P10, P12). Of those without osteoporosis or osteopenia, five had a DXA previously, and the other two would like to have DXA assessment.

All participants had an understanding that osteoporosis caused a weakening of the bones, and most described an association with an increased risk for fracture. Age, female sex and reduced calcium intake were the most commonly reported risk factors.

*[Osteoporosis is when] bones become brittle and break easily* (P5 noOP)

All except one participant had used telemedicine, and two had used it prior to the COVID-19 pandemic. Four participants had used video consults, and the remainder had used telephone consults. Only one participant had used telemedicine specifically for osteoporosis care.

**Fig. 2:**
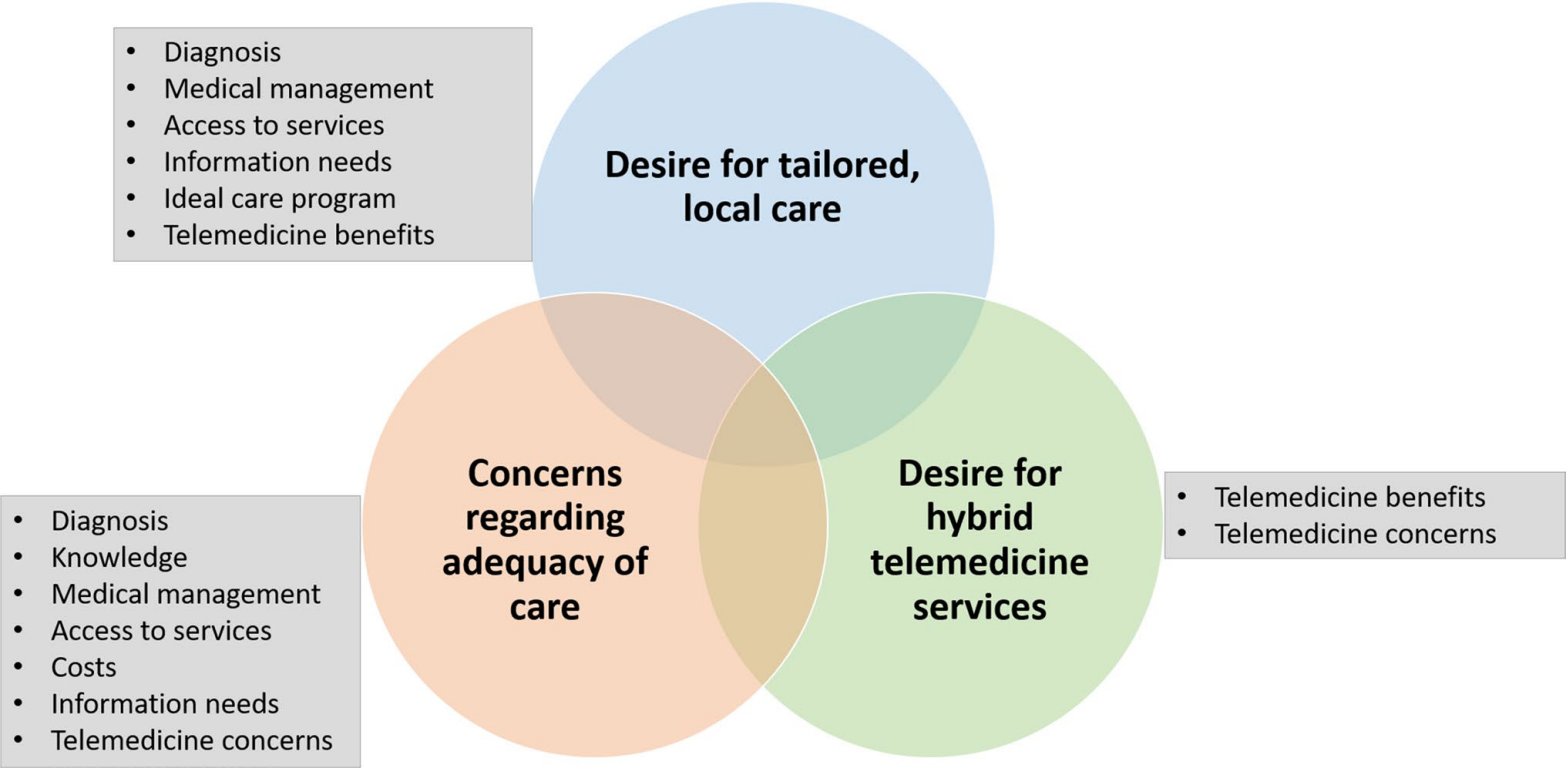
Interview themes and topics. Circles represent themes. Rectangles list the interview topics

Three themes were identified across interview topics: concerns regarding adequacy of care, desire for tailored local care and desire for hybrid in-person or telemedicine appointments (Fig. 2). Additional illustrative quotes can be found in Online Resource 3.

#### Concerns regarding adequacy of care

Of those with osteoporosis, most felt they did not receive sufficient information about osteoporosis at diagnosis, particularly the role of diet and exercise in managing the condition. Even those seeing specialists thought they did not provide sufficient information on lifestyle factors.

*I feel I don’t really know enough to deal with it (P4 OP)*

*[specialists] don’t really go into a lifestyle, living type of thing (P10 OP)*

Participants voiced concerns regarding their medical care from General Practitioners (GPs) and difficulty accessing specialists. GPs were seen as overrun, without enough time to fully deliver the depth of osteoporosis care desired. Participants mentioned receiving different advice from different GPs, between GPs and specialists and between metropolitan and rural clinicians. Other participants received minimal information about the medication they were started on.

*I don’t think a lot of GPs have got the time (P12 OP)*

*I have faith in my GPs, but I think they’re naturally overwhelmed and swamped with many things (P9 noOP)*

*The standard of care is nowhere near as good as it is down there [major city], as far as doctors being educated…I just believe they’re being given different information up here to what [major city] gets (P10 OP)*

Participants reported easy access to a range of allied health services; however, waiting times could be long, and they may not have access to the specific type of service they wanted, such as exercise physiologists.

*We are very blessed with the number of services (P9 noOP)*

*But what I really would like to have seen on that was an exercise physiologist so that I could have some…exercises for me to do that are specific to someone with osteoporosis (P10 OP).*

*They’re not easy to get into and often there’s long waits…it can be three or four months even (P15 OP)*

Awareness of, and access to, DXA was also an issue. Participants wanted greater awareness of the need for screening among doctors and the community. They were also frustrated at having to travel to have a DXA, needing to use the same machine as where they were first diagnosed for ongoing monitoring, as well as the limitations under Medicare (government reimbursement) for DXA intervals.

*For several years I flew back [to South Australia] so that I could be measured on the same [DXA] database and machine every time (P4 OP)*

*I think they should be referring people to have bone density scans more often, and people don’t know to ask for them, and they’ve never had them….so I think there should be a push for that, an education program on asking to have a bone density scan (P10 OP)*

Concern over costs depended on the individual. Most were appreciative of bulk billing services but also willing to pay for services if required. The main concern was not one single cost but the cumulative cost of multiple medical services, especially when people had multiple comorbidities.

*All of the costs are pretty – they’re not excessive but they stretch me (P12)*

#### Desire for tailored, local care

Most participants were interested in more information on osteoporosis, regardless of whether they had osteoporosis or not. However, the preferred type, volume and method of receiving information differed. Participants acknowledged the need for tailored information specific to their needs, with some wanting large amounts of detail, and others preferring not to focus on it.

*Basically knowing what I’m doing, knowing what I need, knowing what it looks like and what it might look like for me and…the important of why it needs to be managed (P15 OP)*

*I don’t really want to focus on it (P2 OP)*

People wanted information available in a variety of formats—written, verbal, internet and in-person education were seen as important. Two-thirds of participants liked the idea of a group education session, and the preference for this was in-person rather than virtually, in the local area. Most agreed this should come from a health professional but did not necessarily need to be a doctor. Participants also wanted a hard copy, written information to which they could refer. This was the preference particularly for those without osteoporosis.

*You get more of a perspective of how everyone else is going and I think more topics that probably are brought up with group [education] (P8 noOP)*

*I wouldn’t want to be travelling a long distance for this (P9 noOP)*

*I actually prefer hard copy pamphlets, booklets, so that I can pick it up and put it down and refer back to [it] (P9 noOP)*

The majority of participants preferred to receive care locally, if possible, although differed in whether they would prefer to have GP-led care or specialist care. The most important factor was expertise in osteoporosis from their clinician, regardless of who this is. Participants wanted a clinician who was thorough, would tailor the treatment to them, and give options, allowing shared, informed decision-making. Allied health was important for some participants, although some felt that they could attend a group allied health session or receive written information, for example, for dietary advice.

*I'd prefer to see someone who was well qualified in the whole picture. GPs tend to have to think of everything, so it might not necessarily be a GP, but it might be a specialist nurse who specialises in that field (P13 noOP)*

*I don’t want a check the box thing, per se…it’s got to be tailored to me, even though it’s a general condition (P1 OP)*

*I'd be willing to travel, but I think it would be lovely if it was someone locally because we've got a big population and it's needed (P13 noOP)*

#### Desire for hybrid in-person or telemedicine appointments

Telemedicine was seen to have multiple benefits, giving better access to services for patients in remote locations. The convenience of telemedicine was obvious, particularly when seeing clinicians in major cities, which can involve long travel times, time off work, stress caused by traffic or parking or reliance on volunteers for transport.

*I think it’s the way of the future (P12 OP)*

*I think that it could well be the opening for fantastic medical provision for remote access people and for people who perhaps have the inconvenience of not having vehicles or access to a vehicle or a disability (P9 noOP)*

However, many participants felt that telemedicine was only suitable for certain issues, such as repeat prescriptions or routine reviews with known doctors. For complex or sensitive issues, mental health, appointments with a new doctor or anything needing a physical examination, participants felt face to face appointments were superior. Participants also acknowledged that privacy may be an issue for some people, particularly if they do not live alone or are dealing with mental health or domestic violence.

*I think it would depend. Look, for instance … I did see specialists in Melbourne and some of them were actually just to catch up after surgery where it would have been great to not have to travel five hours to actually find out that, yeah, we’re happy with you (P15)*

The main concern with telemedicine was the effect on communication and their relationship with their clinician. Participants felt that non-verbal cues may be missed via telemedicine, and incidental issues were less likely to be detected. Telemedicine consults seemed brief, formulaic and lacked the personal connection that many participants feel is important with their doctor. Overall, the vast majority of participants preferred face to face consultations but wanted the ability to choose a telemedicine appointment.

*I think face-to-face is a more personal way of going about it (P11 noOP)*

*When you are face to face you get more visual cues and you get tone of voice and all sorts of, you know, there’s just a difference of being with people person to person (P1 OP)*

## Discussion

This mixed methods study explores osteoporosis among people living in a large regional area of Australia. The survey demonstrated gaps in osteoporosis screening, management and knowledge and a desire for more information about bone health. The qualitative interview findings were also reflected in the survey, with concerns over osteoporosis care, the desire for tailored, individualised and local care and the preference for in-person appointments, with the option of telemedicine.

Our study highlights known gaps in osteoporosis care, with failure to identify a fragility fracture as osteoporosis, and low treatment rates in the surveys, reflected in the interviews as concerns over care [29, 30]. These gaps in care are not unique to Australia or to rural areas; however, the solutions need to be customised. Various models of care to improve osteoporosis outcomes exist, including fracture liaison services, education, orthogeriatric services, universal or targeted screening and exercise. A review of models of care in osteoporosis found mixed efficacy for these programs, and minimal information on implementation characteristics were reported, making it difficult to determine whether existing programs will be suitable and efficacious to scale to new settings [31]. Participants wanted local care if possible, and therefore, approaches such as fracture liaison services, which are mainly located in metropolitan hospitals in Australia currently, will not address this. Virtual fracture liaison services, which have been studied in some countries, may not be desired by the community, as most in our study preferred to see new clinicians in person [32].

Rural and regional areas have poorer access to DXA than metropolitan cities [8, 12]. Our interview participants voiced frustration at having to travel for a DXA but also perceived poor awareness of the need for DXA among the community and clinicians. Aligned with this, over one third of surveyed participants in our study with risk factors for osteoporosis had not had a DXA. Government reimbursed DXA is available in Australia for anyone ≥ 70 years, as well as younger people with specific osteoporosis risk factors. However, unlike other screening programs in Australia, such as for breast cancer or colon cancer, DXA requires a referral from a doctor. Initiatives to improve DXA rates in rural areas include mobile bone densitometry vans; however, a study of a service in rural Queensland still reported lower rates of investigation in more remote areas, suggesting access is not the only barrier [7].

The majority of participants with osteoporosis were managed by their GP; however, GPs have reported knowledge gaps in osteoporosis, with uncertainty around when to start or stop medications, fear of side effects and a desire for more information [12, 33]. A variety of educational programs targeting GPs have been shown to increase DXA and prescribing rates, including electronic reminders, dissemination of guidelines and academic detailing, although most studies also incorporate a patient-directed component [31, 34]. Project ECHO (Extension for Community Healthcare Outcomes) telementoring is one way to offer targeted education and improve osteoporosis care in rural areas [35]. GPs meet with specialists virtually, receive education and training and discuss difficult cases, to upskill them to deliver care for complex patients closer to home. Studies of Bone Health TeleECHO programs in the USA have shown increased confidence in managing osteoporosis among attendees [36]. Improving health professional access to osteoporosis education and research is a key priority of the Australian National Strategic Action Plan for Osteoporosis, and the first Bone Health TeleECHO in Australia launched in 2021 [13]. An updated RACGP/Healthy Bones Australia guideline on the ‘Management of osteoporosis in postmenopausal women and men over 50 years of age’ will also be available in the next year [20].

Interview participants reported that they did not receive enough information about osteoporosis after diagnosis, and this is reflected in the survey osteoporosis knowledge scores, which did not differ between those with or without osteoporosis or fractures. Most people want more information, and there was consensus that information should come from health personnel, so GP practices or community health centres are likely to have a primary role. However, in keeping with previous studies, the desired volume, method, and types of information differed considerably between participants, and so, the approach to education should be individualised [25, 37, 38]. Educational programs are a key component of disease management for a variety of medical conditions and may improve clinical outcomes and self-efficacy; however, outcomes vary considerably between different programs, and the key factors that improve success are unclear [39]. Patient preferences for education may affect these differing outcomes [39, 40]. Clinicians should therefore explore information needs with their patients, and offer tailored information, such as written pamphlets and websites (e.g. Healthy Bones Australia [41]) that patients can refer to.

Most of the survey and interview participants preferred in-person appointments to telemedicine appointments, with the major concerns of telemedicine being communication and the doctor-patient relationship. It is important to note that most participants had used telephone consults, which are now more limited in Australia. Video appointments may address some of the concerns of patients in regards to communication barriers; however, the technology may be more difficult, and internet speeds may be prohibitive for some people [42]. A study of patients attending a metropolitan Australian tertiary osteoporosis clinic via telemedicine found roughly one-quarter preferred in-person appointments, one-quarter preferred telemedicine and the remainder had no preference [43]. Interestingly, confidence with technology was not associated with telemedicine preference. The Endocrine Society have recently published a perspective on the use of telehealth for endocrine conditions [44]. They consider flexibility of appointment type (in-person, telephone or video) important, and the patient preference in regard to this should be strongly considered, with the opportunity to choose their appointment type unless there is strong reason against this. Whether future generations, who have greater familiarity with technology throughout life, will prefer telemedicine appointments is yet to be established.

The three themes identified in the interviews, concerns regarding adequacy of care, desire for tailored local care and desire for hybrid in-person or telemedicine appointments, are interrelated. They all focus on the need for personalised care. Gaps in care, while widespread, are also individual; local services required are personal; and satisfaction with telemedicine depends on the person and clinician involved. Solutions therefore must be thorough and adaptable to the needs of the person.

A strength of our study is the mixed method design and congruent findings from the survey and interviews. However, our study has several limitations. Our participants came from a region within Victoria, Australia, and so findings may not be applicable to other areas. Most of our participants were Australian-born, and only English-speaking participants were included. However, Australian census data suggests that the vast majority of residents of this region are Australian born (80%), and few (6.8%) speak a language other than English at home [45]. A greater proportion of survey respondents had tertiary education compared to the broader region, and while educational attainment was not associated with OKAT scores, it may influence other responses and behaviours. Some survey respondents’ provided postcode was metropolitan, despite stating they lived in a rural area. Survey recruitment was difficult, resulting in a smaller sample size than planned. Recruitment issues may have been due to the COVID-19 pandemic, which prohibited travel of the researchers to the region during recruitment, and meant advertising and interviews had to be performed remotely. This may also reflect a lack of interest in the local community, in general, or due to the pressing importance of the COVID-19 pandemic. Survey respondents were likely therefore more invested or interested in bone health, reflected by relatively high screening rates. Thematic analysis is by its nature subjective, and there may be bias related to the researchers’ knowledge and experience. This analysis was performed by two researchers with different backgrounds, to try and bring diversity to the analysis.

## Conclusions

The over-arching theme emerging from our research is that while gaps exist for osteoporosis care in high fracture risk populations in this region, there is no one-size fits all approach to improving this. Patients have different past experiences of care, access to services and preferences regarding what information and care they want, by whom and how this should be delivered. Health systems, clinicians and care plans must therefore offer a flexible approach, engaging patients to understand their needs and tailoring care to them. More broadly, improving osteoporosis services for regional and rural Australia will require a multi-faceted approach, addressing the needs of the local community and local healthcare providers, as well as health system issues. Co-designed, innovative services are needed to address this and improve outcomes and satisfaction with care.

### Supplementary information

ESM 1 (DOCX 225 kb)

ESM 2 (DOCX 20.5 kb)

ESM 3 (DOCX 18.4 kb)

## Data Availability

Data available on request from corresponding author.
