# Supplementary material for: Bone health in rural Australia: a mixed methods study of consumer needs
Source: Arch Osteoporos. 2023 Oct 14;18(1):127. doi: 10.1007/s11657-023-01333-8 (PMC10576660; doi:10.1007/s11657-023-01333-8)
Supplement: Supplementary file 1 — (DOCX 225 kb) [file 11657_2023_1333_MOESM1_ESM.docx]

Bone health in rural Australia: a mixed methods study of consumer needs. Jones AR, Garth B, Haigh C, Ebeling PR, Teede H & Vincent AJ. *Archives of Osteoporosis.*

Corresponding author: AR Jones, [alicia.jones@monash.edu](mailto:alicia.jones@monash.edu). Monash Centre for Health Research and Implementation, School of Public Health and Preventive Medicine, Monash University, Melbourne, Australia

**Online Resource 1: Survey**

1. What is your age?
2. What is your gender?

- Female
- Male
- Do not identify as either male or female
- Prefer not to answer

1. Were you born in Australia?

- Yes
- No
  - If not, which country were you born in?

1. What ethnicity do you identify as?

- Oceanian (Australian including Indigenous Australian, New Zealander, Pacific Islander)
- North-West European
- Southern and Easter European
- North African and Middle Eastern
- South-East Asian
- North-East Asian
- Southern and Central Asian
- People of the Americas
- Sub-Saharan African
- Other (please specify)

1. Are you of either Aboriginal or Torres Strait islander descent?

- Yes
- No

1. What is your residential postcode?
2. What is the highest level of education you have achieved?

- Primary or middle school / Less that year 12
- Secondary school / Year 12
- Vocational qualification? (Eg apprenticeship)
- Diploma
- Bachelor degree
- Higher degree (master’s, PhD)

1. How often do you use a computer / tablet / iPad / smart-phone

- Daily
- 2-6 times per week
- Weekly
- Monthly
- Less often than monthly
- I do not use any of these devices

The following section refers to your risk factors for osteoporosis.

1. What is your smoking history? (This question refers to cigarettes, cigars and / or pipes)

- Never smoked
- Ex-smoker
- Current smoker less than 1 per day
- Current daily smoker

1. How much alcohol do you, on average, consume? For this question, a standard drink contains 10 grams of alcohol, and is equivalent to 285mL (‘pot’ ‘middy’ ‘handle’) full strength beer / 375mL (stubby / can) mid strength beer / 100mL wine / 30mL spirits

- None
- Less than 1 standard drink per day
- 1 - 2 standard drinks per day
- More than 2 standard drinks per day

1. How much exercise do you do per week? For this question, moderate intensity physical exercise refers to exercises where you are still able to talk while doing them (such as brisk walking, swimming, dancing, raking leaves). Vigorous intensity physical exercise refers to those where you are puffing (such as jogging, cycling, aerobics).

- Less than 75 minutes / 1.25 hours of moderate intensity physical exercise, or less than 35 minutes of vigorous intensity physical activity
- 75-150 minutes / 1.25-2.5 hours of moderate intensity physical exercise, or 35-75 minutes of vigorous intensity physical activity
- 150-300 minutes / 2.5-5 hours of moderate intensity physical exercise, or 75-150 minutes / 1.25-2.5 hours of vigorous intensity physical activity
- More than 300 minutes / 5 hours of moderate intensity physical exercise, or more than 150 minutes / 2.5 hours minutes of vigorous intensity physical exercise

1. Do any of your immediate family members have osteoporosis, or have any of your immediate family members suffered a broken hip?

- Yes
- No

1. Do you have any history of the following conditions? (Tick all that apply)

- Coeliac disease
- Chronic kidney disease
- Chronic liver disease
- Overactive thyroid gland or thyroid cancer
- High parathyroid hormone levels (hyperparathyroidism)
- Low Vitamin D
- Rheumatoid arthritis
- Very high body weight (Eg. body mass index >30, obese)
- Very low body weight (Eg. body mass index <18.5, underweight)
- Epilepsy
- Type 1 or Type 2 Diabetes mellitus
- Malabsorption
- Illness requiring glucocorticoid (steroid such as prednisolone, prednisone, cortisone, hydrocortisone) use for 3 months of longer
- Early menopause (menopause occurring before age 45)
- I have none of these conditions

The following section refers to osteoporosis diagnosis and management.

1. Have you ever had a screening test for osteoporosis (bone densitometry, DXA, heel ultrasound)?

- Yes
- No

1. Have you ever broken / fractured a bone either without any obvious trauma, or after a minor fall such as falling from standing?

- Yes
- No (go to question 17)

1. If Yes, which bone, and how many times have you broken that bone? (Select all that apply)

|  | Yes (tick) | 1 | >1 |
| --- | --- | --- | --- |
| A: Head / face |  |  |  |
| B: Collarbone (clavicle) |  |  |  |
| C: Upper arm / shoulder |  |  |  |
| D: Rib |  |  |  |
| E: Forearm |  |  |  |
| F: Wrist |  |  |  |
| G: Hand / fingers |  |  |  |
| H: Spine (vertebra) |  |  |  |
| I: Pelvis |  |  |  |
| J: Hip |  |  |  |
| K: Thigh bone (femur) |  |  |  |
| L: Lower leg |  |  |  |
| M: Ankle |  |  |  |
| N: Foot / toes |  |  |  |
| O: Other (please specify) |  |  |  |

1. Have you ever been diagnosed with osteoporosis?

- Yes (go to question 18)
- No (go to question 25)

1. If yes, are you or have you previously been on treatment for osteoporosis?

- Yes
- No (go to question 20)

1. If yes, which treatment, and how long for? (Tick all that apply

- Oral Bisphosphonates (Eg. weekly or monthly tablets of risedronate, Actonel, Acris, Risedro, alendronate, Alendro, Alendrobell, Densate, Fonat, Fosamax, Dronalen, ReddyMax)
  - <1 year
  - 1-3 years
  - 4-5 years
  - >5 years
  - I have previously been on this, but am no longer taking this
- Yearly infusion of zoledronic acid (Aclasta, Ostira, Osteovan, Zoledasta)
  - <1 year
  - 1-3 years
  - 4-5 years
  - >5 years
  - I have previously been on this, but am no longer taking this
- Denosumab injections every 6 months (Prolia)
  - <1 year
  - 1-3 years
  - 4-5 years
  - >5 years
  - I have previously been on this, but am no longer taking this
- Teriparatide injections every day (Forteo)
  - <1 year
  - 1-3 years
  - 4-5 years
  - >5 years
  - I have previously been on this, but am no longer taking this
- Hormone replacement therapy
  - <1 year
  - 1-3 years
  - 4-5 years
  - >5 years
  - I have previously been on this, but am no longer taking this
- Calcium supplement
  - <1 year
  - 1-3 years
  - 4-5 years
  - >5 years
  - I have previously been on this, but am no longer taking this
  - Vitamin D supplement<1 year
  - 1-3 years
  - 4-5 years
  - >5 years
  - I have previously been on this, but am no longer taking this

1. Have you ever seen any of the following for management of osteoporosis? (Tick all that apply)

- General Practitioner (go to question 24)
- Specialist non-surgical doctor (Eg Endocrinologist, Rheumatologist) (go to question 21)
- Orthopaedic surgeon
- Naturopath
- Osteopath
- Physiotherapist
- Exercise physiologist
- None of the above

1. If you saw a specialist doctor, how often did you see that specialist?

- Once
- 2-3 times then no further specialist follow-up
- More than 3 times then no further specialist follow-up
- I still see the specialist (at least once per year)
- Other

1. Did you see the specialist in person, or via telemedicine?

- In-person
- Telemedicine – video link
- Telephone

1. If you saw a specialist doctor, did you encounter any problems seeing a specialist for osteoporosis? (Tick all that apply)

- I did not encounter any problems
- Cost of the specialist
- Distance to travel to see a specialist
- Required time off work to see specialist
- Other (please specify)

1. If you did not see a specialist doctor, what is the reason for not seeing a specialist doctor? (Tick all that apply)

- I was not referred to a specialist
- The specialist costs too much
- The distance to see a specialist is too great
- I am unable to get time off work to see a specialist
- I prefer not to see a specialist
- Other (please specify)

1. (For all) Which of the following do you consider important for osteoporosis treatment? If you do not have osteoporosis, please try to imagine that you did?

- Receiving care close to my home
- Seeing a specialist doctor
- Seeing an osteopath
- Seeing a physiotherapist, exercise physiologist
- Seeing a naturopath or other complementary practitioner
- Using telehealth to see doctors or other health professionals
- Access to information to learn more about my condition
  - Online written information
  - Printed, hard copy written information
  - Videos
  - Group education sessions
  - One-on-one education sessions
- Low cost to see a healthcare provider (Eg bulk billing provider)
- Low cost drug treatment
- Flexible times for appointments / ability to choose a time that suits you

Other (please specify)

The following section assesses your knowledge of osteoporosis.

This uses the validated osteoporosis knowledge assessment tool (OKAT) (Winzenberg et al, 2003)

1. Please answer each of the following questions with True, False or Don’t Know.
2. Osteoporosis leads to an increased risk of bone fractures.  True  False  Don’t know
3. Osteoporosis usually causes symptoms ( e.g. pain) before fractures  True  False  Don’t know
   occur.
4. Having a higher peak bone mass at the end of childhood gives **no**  True  False  Don’t know
   protection against the development of osteoporosis in later life.
5. Osteoporosis is more common in men.  True  False  Don’t know
6. Cigarette smoking can contribute to osteoporosis.  True  False  Don’t know
7. White women are at highest risk of fracture as compared to other races.  True  False  Don’t know
8. A fall is just as important as low bone strength in causing fractures.  True  False  Don’t know
9. By age 80, the majority of women have osteoporosis.  True  False  Don’t know
10. From age 50, most women can expect at least one fracture before they  True  False  Don’t know
    die.
11. Any type of physical activity is beneficial for osteoporosis.  True  False  Don’t know
12. It is easy to tell whether I am at risk of osteoporosis by my clinical risk  True  False  Don’t know
    factors.
13. Family history of osteoporosis strongly predisposes a person to  True  False  Don’t know
    osteoporosis.
14. An adequate calcium intake can be achieved from two glasses of  True  False  Don’t know
    milk a day.
15. Sardines and broccoli are good sources of calcium for people who  True  False  Don’t know
    cannot take dairy products.
16. Calcium supplements alone can prevent bone loss.  True  False  Don’t know
17. Alcohol in moderation has little effect on osteoporosis.  True  False  Don’t know
18. A high salt intake is a risk factor for osteoporosis.  True  False  Don’t know
19. There is a small amount of bone loss in the ten years following  True  False  Don’t know
    the onset of menopause.
20. Hormone therapy prevents further bone loss at any age after menopause.  True  False  Don’t know
21. There are no effective treatments for osteoporosis available in Australia.  True  False  Don’t know

The following questions refer to telemedicine, particularly during the Covid-19 pandemic

1. Have you used telemedicine (telephone or telehealth (video)) to see a doctor during the Covid-19 pandemic?

- Yes
- No (go to Q31)

1. If yes, how many times have you used telemedicine for a consultation? (free text)
2. Did you use
   - Telephone
   - Video link
3. Was this appointment to see a:

- General Practitioner?
- Specialist (specify type)
- Allied health (Eg physiotherapist, dietician)

1. Have you used telemedicine before the Covid-19 pandemic?

- Yes
- No (go to question 35)

1. To what extent do you agree with the following? (options strongly disagree, somewhat disagree, neither disagree or agree, somewhat agree, strongly agree))
   - Instructions provided to me by the clinic prior to the appointment were easy to understand
   - The telemedicine system was easy to use
   - The quality of audio / video connection was good
   - I am generally confident with computers / technology
   - The quality of care I received was the same as if it had been an in-person visit
   - It was easy to access any paperwork after the clinic visit (including blood tests, X-ray, medical certificates, prescriptions)
   - Using telemedicine was convenient
   - The telemedicine visit adequately addressed my needs
2. Comparing telemedicine to a traditional, in-person medical appointment, how would you rate each of the following? (options telemedicine better, in-person better, no difference, not sure)

- Convenience
- Time taken to travel for the visit
- Waiting time for the visit
- Persoonal connection I feel with my doctor
- Amount of time I spend with my doctor
- Overall experience

1. How happy would you be to use telemedicine again, if you could choose to after the Covid-19 pandemic has ended?
   - Definitely would
   - Probably would
   - Probably would not
   - Definitely would not
   - Not sure
2. What are the reasons for not using telemedicine? (tick all that apply)
   - I have not needed to visit a health professional during this time (go to Q37)
   - My health professional does not offer telemedicine (go to Q37)
   - I do not have the ability (phone or internet connection) to use telemedicine at my place of residence (go to Q37)
   - I prefer to see a health professional in-person (go to Q38)
   - Other (please specify)
3. If you did need to visit a doctor and you could use telemedicine, would you use it?
   - Definitely would
   - Probably would
   - Probably would not
   - Definitely would not
   - Not sure
4. (If answer d to Q36) What are the reasons for preferring to see a doctor in-person? (tick all that apply)
   - I am concerned about the privacy of my medical information when using telemedicine
   - I am concerned about preparing myself or my surroundings when using telemedicine
   - I am concerned about not getting adequate treatment if I have a telemedicine visit
   - I am concerned about not being able to show my doctor a physical problem
   - I find it more difficult to talk with my doctor when using telemedicine
   - I am concerned about being able to use the telemedicine system
   - Other (please specify)
5. (for all) Regarding concerns or worries about telemedicine, to what extent do you agree with the following? (options strongly disagree, somewhat disagree, neither disagree or agree, somewhat agree, strongly agree))
   - I am concerned about the privacy of my medical information using telemedicine
   - I am concerned that my phone or internet connection will not be good enough for telemedicine
   - I do not have a suitable location for using telemedicine (eg privacy at home, ability to be away from work)
   - I find it difficult to feel connected to my doctor via telemedicine
   - I am concerned about not getting adequate treatment if I have a telemedicine visit
   - Other (please specify)
