# Supplementary material for: Bone health in rural Australia: a mixed methods study of consumer needs
Source: Arch Osteoporos. 2023 Oct 14;18(1):127. doi: 10.1007/s11657-023-01333-8 (PMC10576660; doi:10.1007/s11657-023-01333-8)
Supplement: Supplementary file 2 — (DOCX 20.5 kb) [file 11657_2023_1333_MOESM2_ESM.docx]

Bone health in rural Australia: a mixed methods study of consumer needs. Jones AR, Garth B, Haigh C, Ebeling PR, Teede H & Vincent AJ. *Archives of Osteoporosis.*

Corresponding author: AR Jones, [alicia.jones@monash.edu](mailto:alicia.jones@monash.edu). Monash Centre for Health Research and Implementation, School of Public Health and Preventive Medicine, Monash University, Melbourne, Australia

**Online Resource 2. Semi-structured interview schedule**

Hello, thank-you for volunteering your time today. My name is <insert researcher’s name here> and I am one of the research investigators.

This interview will assess your opinions on osteoporosis, and your preferences for osteoporosis care. The information will be used to inform the development of an osteoporosis care program. It will take approximately 10-15 minutes.

I will be recording our discussion and taking notes, but your answers and names will not be shared with people outside the research team. All information will be recorded anonymously, and any information published will anonymous. If you do not feel comfortable answering any questions, please tell me and we will move on to the next question.

By taking part, you consent to the anonymous publication of data.

Before I begin, do you have any questions? Are you willing to proceed with the interview?

I will start with some initial screening questions, to determine which interview schedule to use.

| Are you male, female, or would you prefer not to answer? |  |
| --- | --- |
| How old are you? |  |
| Would you describe where you live as in a major city, regional city, rural or remote area? |  |
| Are you currently working? | If so, in what area? |
| Have you ever been diagnosed with osteoporosis or osteopenia? |  |

**Option 1: Participants with osteoporosis or osteopenia diagnosed**

| **Questions** | **Prompts** |
| --- | --- |
| What do you know about osteoporosis? | What do you think osteoporosis is?  Do you know any risk factors for osteoporosis? |
|  | |
| (Break for researcher to state the following)  From a medical perspective, osteoporosis refers to a condition where bone strength weakens – bones become more fragile and so break more easily. Osteoporosis is more common in women after menopause, but also affects males, younger people, and people with other medical conditions or taking certain medications.  Osteoporosis is managed with lifestyle programs such as exercise, Vitamin D and calcium supplementation, and medications. | |
| When were you diagnosed with osteoporosis? |  |
|  | |
| How were you diagnosed with osteoporosis? | - Who made the diagnosis (GP, specialist) - How was this made (Eg screening, after a fracture) |
|  | |
| When you were diagnosed with osteoporosis, did you receive enough information to understand what it is, why it occurs and what can be done to manage / treat it? |  |
|  | |
| Have you received any treatment for osteoporosis in the past or currently? This includes medications taken by mouth, injections, specific exercise programs, specific diet advice. | If previous medication why was this stopped or changed?  If received medical treatment: why was this specific treatment chosen? |
|  | |
| Have you experienced any difficulty in managing your osteoporosis? | Consider   - Access to educational resources - Costs of medical therapy - Costs of seeing doctors / allied health - Access to doctors / allied health / investigations - Travel time to access above |
|  | |
| Have you seen a doctor for osteoporosis using telemedicine?  (If not) Have you seen a doctor for any condition using telemedicine? | Clarify whether this was during the Covid-19 pandemic or not  Clarify video or telephone |
|  | |
| (If have used telemedicine) What was your overall satisfaction with using telemedicine? | Consider   - Technical issues, audio / video quality, connection speed - Connection / rapport with clinician - Quality of care received - Ability to access scripts, pathology tests - Convenience: time saved in travel., waiting times, ability to have appointment at time that suits you - Cost |
|  | |
| Do you have any concerns about using telemedicine? | Consider   - Privacy - Technology issues - Quality of care received - Relationship with clinician |
|  | |
| (if have not used telemedicine)  Would you consider using telemedicine in the future? Why / why not?  Do you think telemedicine would be a good option for osteoporosis? |  |
|  | |
| I would like you to consider now your ‘perfect’ care program for osteoporosis. In terms of medical care by your GP or a specialist doctor, what would this consultation look like? | For example, think about *where* you would like to see a health professional, both initially and at follow-up, *how* often you would like to see them, the cost of this, whether you would prefer to see them in person or have communication via phone or email. |
|  | |
| Would any other healthcare professionals be useful in assisting to manage your osteoporosis? Have you experienced any barriers in accessing these services? How would you like to see these people? | For example, physiotherapists, exercise physiologist, dietician, osteopath, complementary medicine practitioner? In person vs telemedicine? |
|  | |
| If there was an educational program to find out more about osteoporosis, what would your ideal program look like? | How would you like to receive information? (written hard copy, electronic, verbal, videos)  Who would you like to provide the education? Would you prefer a doctor, a nurse, a peer who has had training?  Would prefer education in a group or alone?  Where would you like the education to occur? Would you prefer it at home, at a community place such as a library, at your local GP clinic?  Would you prefer to have a once-off education, or multiple sessions?  If not, why is this? |

**Option 2: Participants without osteoporosis or osteopenia**

| **Questions** | **Prompts** |
| --- | --- |
| What do you know about osteoporosis? | What do you think osteoporosis is?  Do you know any risk factors for osteoporosis? |
|  | |
| (Break for researcher to state the following)  From a medical perspective, osteoporosis refers to a condition where bone strength weakens – bones become more fragile and so break more easily. Osteoporosis is more common in women after menopause, but also affects males, younger people, and people with other medical conditions or taking certain medications.  Osteoporosis is managed with lifestyle programs such as exercise, Vitamin D and calcium supplementation, and medications. | |
| If there was an educational program to find out more about osteoporosis, would you be interested in taking part in this? | How would you like to receive information? (written hard copy, electronic, verbal, videos)  Who would you like to provide the education? Would you prefer a doctor, a nurse, a peer who has had training?  Would prefer education in a group or alone?  Where would you like the education to occur? Would you prefer it at home, at a community place such as a library, at your local GP clinic?  Would you prefer to have a once-off education, or multiple sessions?  If not, why is this? |
|  | |
| Would you like to find out more about your personal risk of osteoporosis? | If so, how?  Would you use an online tool or calculator?  Would you have a risk assessment with a doctor or a nurse?  Would you go and have a blood test or X-Ray?  If not, why is this? |
|  | |
| I would like you to imagine that you needed to see a doctor for osteoporosis. What would the ‘ideal’ consultation or program look like? | For example, think about *where* you would like to see a health professional, both initially and at follow-up, how often you would like to see them, the cost of this, whether you would prefer to see them in person or have communication via phone or email. |
|  | |
| Do you think any other healthcare providers would be useful to assist in diagnosing or managing osteoporosis? If yes, how would you like to see them? | For example, physiotherapist, pharmacist, exercise physiologist, dietician, osteopath, complementary medicine practitioner? |
|  | |
| The following questions refer to the Covid-19 pandemic | |
| Have you seen a medical professional during the Covid-19 pandemic? Was this in-person or using telemedicine? | Clarify video or telephone |
|  | |
| (If have used telemedicine) What was your overall satisfaction with using telemedicine? | Consider   - Technical issues, audio / video quality, connection speed - Connection / rapport with clinician - Quality of care received - Ability to access scripts, pathology tests etc - Convenience: time saved in travel., waiting times, ability to have appointment at time that suits you - Cost |
|  | |
| Do you have any concerns about using telemedicine? | Consider   - Privacy - Technology issues - Quality of care received - Relationship with clinician |
|  | |
| (if have not used telemedicine)  Would you consider using telemedicine in the future? Why / why not? |  |
