# Supplementary material for: Bone health in rural Australia: a mixed methods study of consumer needs
Source: Arch Osteoporos. 2023 Oct 14;18(1):127. doi: 10.1007/s11657-023-01333-8 (PMC10576660; doi:10.1007/s11657-023-01333-8)
Supplement: Supplementary file 3 — (DOCX 18.4 kb) [file 11657_2023_1333_MOESM3_ESM.docx]

Bone health in rural Australia: a mixed methods study of consumer needs. Jones AR, Garth B, Haigh C, Ebeling PR, Teede H & Vincent AJ. *Archives of Osteoporosis.*

Corresponding author: AR Jones, [alicia.jones@monash.edu](mailto:alicia.jones@monash.edu). Monash Centre for Health Research and Implementation, School of Public Health and Preventive Medicine, Monash University, Melbourne, Australia

**Online Resource 3: Supplementary Tables**

**Supplementary Table 1: Characteristics of participants in qualitative study**

| Participant | Sex | Age | Residential location |
| --- | --- | --- | --- |
| 1 (OP) | F | 77 | rural |
| 2 (OP) | F | 87 | rural |
| 3 (noOP) | F | 69 | regional |
| 4 (OP) | F | 74 | rural |
| 5 (noOP) | F | 59 | rural |
| 6 (noOP) | F | 64 | regional |
| 7 (OP) | F | 75 | rural |
| 8 (noOP) | F | 73 | rural |
| 9 (noOP) | F | 60 | regional |
| 10 (OP) | F | 63 | rural |
| 11 (noOP) | F | 66 | rural |
| 12 (OP) | M | 80 | regional |
| 13 (noOP) | F | 75 | rural |
| 14 (OP) | F | 73 | rural |
| 15 (OP) | F | 57 | regional |

OP: diagnosis of osteoporosis or osteopenia; noOP: no diagnosis of osteoporosis or osteopenia; F: female; M: male. Residential location was self-reported.

**Supplementary Table 2: Additional illustrative quotes from interviews.**

| **Interview topic** | **Theme** | **Illustrative Quotes** |
| --- | --- | --- |
| Knowledge | Concern regarding adequacy of care | “I don’t think I’ve had anything in the way of dietary advice” (P1 OP) |
|  |  | “I feel like my knowledge is pretty good” (P6 noOP) |
| Diagnosis | Concern regarding adequacy of care | “it was probably assumed that I knew that I had osteoporosis” (P1 OP) |
|  |  | “Nothing, nothing at all, just basically a fleeting comment” (P15 OP) |
| Medical management | Concern regarding adequacy of care | “The medical management could have been better” (P1 OP) |
|  |  | “I’m not sure of the quality of the specialists that come to regional areas…whether the good doctors come up” (P10 OP) |
|  |  | “I certainly would not get treatment from anyone up here for my osteoporosis” (P10 OP) |
| Access to services | Desire for tailored, local care | “I’m lucky…where I live, we’ve got several good physios here” (P1 OP) |
|  | Concern regarding adequacy of care | “They’re so stretched to the limit, it’s hard to get in there” (P12 OP) |
|  | Concern regarding adequacy of care  Desire for tailored, local care | “[my endocrinologist is] very hard to get an appointment with...in fact, nearly impossible” (P14 OP) |
| Access to services - DXA | Concern regarding adequacy of care  Desire for tailored, local care | “I would like to be able to access locally a bone density scanner that is up to date”. (P10 OP) |
|  | Concern regarding adequacy of care | “So they reckon Medicare will only do you every 5 years” (P14 OP) |
| Costs |  | “I think if you get the right advice it’s [the cost] worth it definitely” (P8 noOP) |
|  |  | “I’m very fortunate to be financially able to do what I require and what is necessary” (P9 noOP) |
|  | Concern regarding adequacy of care | “I don’t have to be bulk billed, necessarily, but I do look at some of their costs” (P9) |
| Information needs | Desire for tailored, local care | [regarding group education] “Then you could sort of ask questions and people ask questions that you don’t think about and yet get answers to something you haven’t thought about” (P3 no OP) |
|  |  | “It’s nice to talk to people who are going through what you’re going through” (P10 OP) |
|  |  | “It has to be convenient” (P12 OP) |
|  | Concern regarding adequacy of care  Desire for tailored, local care | “The thing is, you don’t know what you don’t know” (P10 OP) |
| Ideal care program – medical care | Desire for tailored, local care | “I want somebody who is thorough and who will have a wider view than just “get her on calcium and vitamin D, she should be right”” (P1 OP) |
|  |  | “I want the right person for the job. I want a person who’s able to refer on if necessary to more expertise “ (P1 OP) |
|  |  | “Just here locally…my local GP or somewhere local or your community health area” (P3 noOP) |
|  |  | “A GP would be fine as long as they knew what they were talking about” (P4 OP) |
|  |  | “We'd travel to do certain things as long as it wasn't too often basically” (P8 noOP) |
|  |  | “I think locally as long as they have the expertise” (P15 OP) |
|  | Concern regarding adequacy of care  Desire for tailored, local care | “I wouldn't use a GP for my osteoporosis, because I don't know if they're trained enough” (P10 OP) |
| Ideal care – allied health | Desire for tailored, local care | “Look I think a one-off dietician thing but I think that's something that you could probably get something in the mail and give you a bit of an idea what's good to eat and what's not” (P3 noOP) |
|  |  | “I'm thinking of my community here that a number of people would be unable to go one-on-one with a physio necessarily, but they could attend a community health centre network group.” (P9 noOP) |
| Telemedicine benefits | Desire for hybrid in-person and telemedicine appointments | “So much easier to just have a phone call in the comfort of your own home” (P3 noOP) |
|  |  | “Absolutely fantastic. The fact that you don’t have to leave your home and you don’t have to actually…there’s usually quite a long waiting time when you go to the clinic” (P10 OP) |
|  |  | “If they said I had to take myself or [wife] to [major city] at the moment I would go into a major meltdown because of the traffic” (P12 OP) |
|  |  | “Just to go in and get prescriptions it’s crazy to take his time, there must be a better way to do it” (P12 OP) |
|  |  | “It’s ok with the GP because I’ve known her for a while” (P13) |
| Telemedicine concerns | Concern regarding adequacy of care  Desire for hybrid in-person and telemedicine appointments | “You don't seem to talk to them as much. It's not as personal. …Particularly if there's something that concerns one or one feels a bit emotional about you can't sort of open up on the phone or I couldn't” (P2, OP) |
|  |  | “He answers what I ask and deals with the matters I bring up although it's not the same quality” (P2 noOP) |
|  |  | “Because of mental health and various other issues, so much can be hidden and glossed over and purposefully disguised by patients” (P9 noOP) |
|  |  | “Being in a room with a person is a totally different experience to talking to them on the phone” (P13 noOP) |
|  |  | “I have a major problem with using telemedicine in all fields, because for instance, my old colleagues who are trying to pick up things like domestic violence, it's amazing what you pick up when you're in the room with a person” (P13 noOP) |
|  |  | “Look, it’s great and it has a purpose. I saw in some areas, like that person one-on-one I feel like I’m held more responsible when I actually see someone face-to-face” (P15 OP) |
